# Use of antiseptic for cord care and its association with neonatal mortality in a population-based assessment in Bihar State, India

**DOI:** 10.1136/bmjopen-2016-012436

**Published:** 2017-01-25

**Authors:** Rakhi Dandona, Priyanka S Kochar, G Anil Kumar, Lalit Dandona

**Affiliations:** 1 Public Health Foundation of India, New Delhi, India; 2 Institute for Health Metrics and Evaluation, University of Washington, Seattle, Washington, USA

**Keywords:** cord care, gentian violet, India, Neonatal mortality

## Abstract

**Objectives:**

Dry cord care is recommended for all births by the Health Ministry in India. We report prevalence of antiseptic cord care in the context of neonatal mortality in the Indian state of Bihar.

**Design:**

Population-based cross-sectional study with multistage stratified random sampling.

**Setting:**

Households in 1017 clusters in Bihar.

**Participants:**

A representative sample of 12 015 women with a live birth in the last 12 months were interviewed from all 38 districts of Bihar (90.7% participation) in 2014.

**Primary and secondary outcome measures:**

Use of antiseptic cord care at birth and its association with neonatal mortality using multiple logistic regression.

**Results:**

Topical application of any material on cord was reported by 6534 women (54.4%; 95% CI 53.5% to 55.3%). Antiseptic cord care prevalence was 49.7% (95% CI 48.8% to 50.6%), the majority of which was gentian violet (76.4%). The odds of antiseptic use for cord care were higher in facility births (OR 1.46; 95% CI 1.27 to 1.69) and for deliveries by a qualified health provider (OR 1.44; 95% CI 1.26 to 1.66), but were lower for births that occurred before the expected delivery date (OR 0.77; 95% CI 0.61 to 0.96). A total of 256 (2.1%) newborns died during the neonatal period. The odds of neonatal death were significantly higher for live births with no reported antiseptic use (OR 1.53; 95% CI 1.18 to 1.99), and this association persisted when live births in health facilities were considered separately.

**Conclusions:**

Even though dry cord care is recommended by health authorities in India, half the women in this study reported use of antiseptic for cord care mainly with gentian violet; and its use had beneficial effect on neonatal mortality. These findings suggest that the application of readily available gentian violet for cord care in less developed settings should be assessed further for its potential beneficial influence on neonatal mortality.

**Strengths and limitations of this study**

- This is a large population-based cross-sectional representative sample of live births in the Indian state of Bihar with high neonatal mortality.
- The study documented information on antiseptic use for cord care and other variables that could possibly influence neonatal mortality through interviews.
- The timing of antiseptic use, who applied the antiseptic and incidence of omphalitis were not documented.

## Introduction

Neonatal deaths accounted for 42% of the under-5 deaths worldwide in 2013.^1^ Infections contribute to about one-third of the annual neonatal deaths in developing countries.^2^ Umbilical cord infection (omphalitis), a risk factor for neonatal sepsis, is an important public health problem in low-resource settings where home deliveries are common and neonatal mortality is high.^3^ ^4^ The WHO has for some time promoted dry umbilical cord care for newborn infants because of the likelihood of harmful practices in regions with high neonatal infections, and due to the lack of direct evidence to recommend the widespread use of topical antimicrobials on the umbilical cord stump until the recent community trials of chlorhexidine.^5–9^ Two recent Cochrane reviews concluded that there was evidence to suggest that topical application of chlorhexidine to the umbilical cord reduces neonatal mortality and omphalitis in community and primary care settings in developing countries, but evidence to support the application of an antiseptic to umbilical cord in hospital settings compared with dry cord care was insufficient.^7^ ^10^ ^11^ Currently, the WHO guidelines on postnatal care recommend chlorhexidine application to the umbilical cord stump during the first week of life for home births in regions with high neonatal mortality, and clean dry cord care for facility births and for home births in low neonatal mortality settings.^12^ ^13^

Despite tremendous gains made in maternal and child health since 1990, India still accounts for a considerable proportion of the global neonatal deaths,^1^ with nearly a quarter of neonatal deaths in India being attributable to neonatal sepsis.^14^ ^15^ The Ministry of Health and Family Welfare in India also recommends dry cord care for all births.^16^ We had an opportunity to assess cord care practices in the Indian state of Bihar which is the third most populous state in India with a population of over 100 million.^17^ The Ananya programme, funded by the Bill & Melinda Gates Foundation, is being implemented in the state of Bihar with the goal of reducing child and maternal mortality, fertility and undernutrition.^18^ We have previously reported a relatively high neonatal mortality rate of 32.2 (95% CI 27.6 to 36.9) per 1000 live births in Bihar in 2012.^19^ In the background of high neonatal mortality and given the data available on cord care practices, we explored the use of antiseptic cord care at birth and its association with neonatal mortality in a population-based cross-sectional survey conducted in 2014 across all districts of Bihar.

## Methods

Detailed methodology for the baseline survey performed in 2012 is available in the previously published reports on neonatal mortality and stillbirth estimates for the state of Bihar.^19^ ^20^ Another survey was conducted from January to March 2014 using the sampling framework of the previous survey performed in 2012.^19^ ^20^ This paper reports on data from the survey performed in 2014. Briefly, we used a multistage stratified random sampling approach to obtain a representative sample of 772 rural and 245 urban clusters, a total of 1017 clusters of about 75–150 households across all the 38 districts of Bihar. The sample was based on having 15 390 live births in the sampled clusters which would give reasonable power to detect a significant change in neonatal mortality rate over the intervention period of 5 years.

A household was defined as people eating from the same kitchen, and the number of households within a particular cluster was obtained from the baseline survey. Information was collected on all pregnancy outcomes experienced by women aged 15–49 years in the last 12 months. A birth was considered live if the newborn had breathed or cried or moved at birth.^21^ All women who reported having had a live birth in the last 12 months were eligible for interview. Written informed consent was obtained for participation in the survey. Illiterate persons provided the right thumb impression in lieu of signature for consent. For participating women, detailed interviews were conducted by trained interviewers to document information on socio-demographic background, birth history and usage of maternal and childcare services for the birth in the last 12 months. If a woman reported multiple births from one pregnancy, the details of the infant born first were recorded. Women were asked if any material was applied to the cord of neonate after cutting at birth and what it was. This was an open-ended question, and the interviewer marked the appropriate precoded category based on the response given by the participating women. The questionnaire included local terminologies for the material expected to be in use in this population. If a response code did not exist for the material reported, then it was documented in full. The questionnaire did not document who applied the antiseptic and where it was applied. Data were entered directly in a computer by the interviewers, which was scrutinised to detect and correct errors using the procedures standardised in the baseline study to meet the data quality of the midline study as well. About 30% of the data were collected by the interviewers under direct supervision and an additional 5% of the interviews were checked by the supervisors by visiting the respondent again.^19^ ^20^

Data were analysed using STATA V.11.2 software (Stata Corp, USA). The main exposure of interest was use of antiseptic for cord care which was defined as application of any of the following materials to the cord: gentian violet, antiseptic powder/ointment, alcohol, boric powder or chlorhexidine. We report the prevalence of use of antiseptic for cord care among live births in the last 12 months in the state of Bihar. We examined a variety of individual-level and delivery-related associations with use of antiseptic for cord care using multiple logistic regression based on our understanding of the local context related to this. In addition, we explored the association of use of antiseptic for cord care with neonatal mortality defined as death occurring within the first 28 days of life, using multiple logistic regression analysis in which other relevant individual, antenatal, delivery and postnatal variables that could potentially influence neonatal mortality were adjusted for. Household Wealth Index, one of the variables in the model, was calculated as in the National Family Health Survey.^22^ In both the multiple logistic regression models, correlation between the variables being considered for the model was assessed using the Pearson correlation and collinearity diagnostic test in SPSS following which two variables were not included (birth order of the child and caste of the mother). The effect of each category of a multicategorical variable was assessed by keeping the first or the last category as reference, and all the variables were introduced simultaneously into the model. The ORs are presented with 95% CIs. χ^2^ test is reported where relevant to assess significant univariate associations; p<0.05 was considered significant.

## Results

In the 103 551 households enumerated in the sampled clusters, 13 249 women were identified with a live birth in the last 12 months, of whom 12 015 (90.7%) participated, 624 (4.7%) were out of station for an extended period, 565 (4.3%) were not available due to other reasons and 45 (0.3%) refused participation.

Of the 12 015 women with a live birth in the last 12 months who participated, topical application of any material after cutting and tying of the cord was reported by 6534 (54.4%; 95% CI 53.5% to 55.3%), 4930 (41%) reported that nothing was applied and 551 (4.6%) did not know if anything was applied. A total of 5969 women reported application of any antiseptic material on cord of the neonate at birth, giving a 49.7% (95% CI 48.8% to 50.6%) prevalence of the use of antiseptic for cord care. Of the women who had reported antiseptic application on the cord, gentian violet was reported by 4560 (76.4%), antiseptic ointment by 1209 (20.2%), alcohol by 138 (2.3%), boric acid by 58 (0.97%) and chlorhexidine was reported only by four women (0.07%). Overall, 565 (4.7%) women reported use of potentially harmful substances such as sindoor (cosmetic powder), ghee (a type of oil) and cow dung.

Among the 3242 births at home, prevalence of the use of antiseptic for cord care was 36.6% (95% CI 35.0% to 38.3%). Majority of the home births were attended by an unqualified healthcare provider (3006; 92.7%), of which 1064 (35.4%) reported use of antiseptic. In comparison to the 235 (7.3%) deliveries at home that were attended by a qualified healthcare provider, a higher proportion reported use of antiseptic on the cord (124, 52.8%; p<0.001). The use of harmful substances on the cord was higher in home births, with 312 (9.6%) reporting this use, as compared with only 253 (2.9%) of the facility births (p<0.001).

Using multiple logistic regression (table 1), the odds of use of antiseptic for cord care were significantly higher for facility births (OR 1.46; 95% CI 1.27 to 1.69) as compared with the births at home, with private health facilities having higher odds of antiseptic use than public health facilities. Deliveries by a qualified health provider had significantly higher reporting of the use of antiseptic for cord care (OR 1.44; 95% CI 1.26 to 1.66), with the odds being highest for deliveries conducted by auxiliary nurse midwife (ANM) (OR 1.92, 95% CI 1.59 to 2.32) as compared with unqualified health providers. Women with live births before the expected delivery date were significantly less likely to report use of antiseptic for cord care (OR 0.77; 95% CI 0.61 to 0.96). The odds of the use of antiseptic for cord care were higher if the birth order of the child was three or lower (OR 1.15, 95% CI 1.04 to 1.26).

**Table 1:** Association of select variables with reported use of antiseptic for cord care among women who reported a live birth in the last 12 months in the Indian state of Bihar using multiple logistic regression

Variable	Category	Total N=12 015 (% of total)	Number reported use of antiseptic for cord care (% of total)	OR for use of antiseptic for cord care (95% CI)
Birth order of the child*	1–3	9521 (79.2)	4868 (51.1)	1.15 (1.04 to 1.26)
	>3	2494 (20.8)	1101 (44.1)	1.00
Maternal schooling*	No schooling	6089 (50.7)	2844 (46.7)	1.00
	Any schooling	5926 (49.3)	3125 (52.7)	1.05 (0.97 to 1.14)
Place of residence*	Urban	2220 (18.5)	1226 (55.2)	1.17 (1.06 to 1.29)
	Rural	9795 (81.5)	4743 (48.4)	1.00
Caste†	Forward caste	3316 (27.6)	1588 (47.9)	1.00
	Others	8699 (72.4)	4381 (50.4)	1.14 (1.05 to 1.24)
Place of delivery*	Home	3242 (27.0)	1188 (36.6)	1.00
	Public health facility	6586 (54.8)	3440 (52.2)	1.40 (1.21 to 1.62)
	Private health facility	2187 (18.2)	1341 (61.3)	2.01 (1.70 to 2.39)
Time of delivery‡	On time or late	11 657 (97.0)	5809 (49.8)	1.00
	Early	358 (3.0)	160 (44.7)	0.77 (0.61 to 0.96)
Healthcare provider who cut the umbilical cord*§	Doctor	562 (4.7)	282 (50.2)	1.13 (0.92 to 1.40)
	Nurse	6858 (57.1)	3762 (54.9)	1.37 (1.19 to 1.57)
	ANM/SBA	813 (6.8)	499 (61.4)	1.92 (1.59 to 2.32)
	Unqualified	3662 (30.5)	1383 (37.8)	1.00

*χ^2^ test for significance: p<0.001.

†χ^2^ test for significance: p=0.015; others include backward caste, scheduled caste and scheduled tribe.

‡χ^2^ test for significance: p=0.055; time of delivery as defined by the respondent.

§Data missing for 120 live births.

ANM, auxiliary nurse midwife; SBA, skilled birth attendant.

A total of 256 (2.1%; 95% CI 1.89% to 2.40%) newborns had died during the neonatal period among the sample that provided detailed interviews. The live births for whom nothing was applied for cord care were significantly more likely to die during the neonatal period (OR 1.53; 95% CI 1.18 to 1.99) as compared with those who reported use of antiseptic on the cord, after adjusting for other socio-demographic and healthcare variables in the multiple logistic regression analysis (table 2). This association was also seen when live births in health facilities were considered separately (OR 1.52; 95% CI 1.12 to 2.06). Among the 358 preterm babies in this study who were born with a gestation period of 8 months or less, 160 (44.7%) reported use antiseptic for cord care and the rest did not. Nine (5.6%) of those for whom antiseptic use was reported had died as compared with 27 (13.6%) of those for whom antiseptic use was not reported (p=0.012).

**Table 2:** Association of select variables with neonatal mortality among women who reported a live birth in the last 12 months in the Indian state of Bihar using multiple logistic regression

		Overall (N=12 015)			Only institutional deliveries (N=8773)		
Variable	Category	Total (% of total)	Number of neonatal deaths (% of total)	OR for neonatal death (95% CI)	Total (% of total)	Number of neonatal deaths (% of total)	OR for neonatal death (95% CI)
Maternal age at birth (years)*	15–19	460 (3.8)	16 (3.5)	1.85 (1.06 to 3.20)	361 (4.1)	13 (3.6)	2.01 (1.08 to 3.74)
	20–24	5087 (42.3)	126 (2.5)	1.41 (1.07 to 1.84)	3910 (44.6)	95 (2.4)	1.43 (1.04 to 1.97)
	25–34	5892 (49.0)	106 (1.8)	0.73 (0.35 to 1.51)	4148 (47.3)	70 (1.7)	0.48 (0.15 to 1.55)
	35+	576 (4.8)	8 (1.4)	1.00	354 (4.0)	3 (0.9)	1.00
Maternal schooling†	No schooling	6089 (50.7)	134 (2.2)	1.13 (0.86 to 1.50)	3965 (45.2)	80 (2.0)	1.00 (0.72 to 1.38)
	Any schooling	5926 (49.3)	122 (2.1)	1.00	4808 (54.8)	101 (2.1)	1.00
Sex of the neonate‡	Boy	6464 (53.8)	152 (2.4)	1.25 (0.97 to 1.61)	4696 (53.5)	104 (2.2)	1.17 (0.86 to 1.58)
	Girl	5549 (46.2)	104 (1.9)	1.00	4075 (46.5)	77 (1.9)	1.00
Wealth index quintile for the household§	Quintile 1–4	9717 (81.0)	218 (2.2)	1.40 (0.95 to 2.07)	6761 (77.2)	148 (2.2)	1.40 (0.93 to 2.10)
	Quintile 5	2286 (19.0)	38 (1.7)	1.00	2003 (22.8)	33 (1.7)	1.00
Place of delivery¶	Home	3242 (27.0)	75 (2.3)	1.00			
	Public health facility	6586 (54.8)	128 (1.9)	1.24 (0.90 to 1.70)			
	Private health facility	2187 (18.2)	53 (2.4)	1.53 (1.00 to 2.34)			
Maternal complication during pregnancy**	No	9655 (80.4)	185 (1.9)	1.00	6903 (78.7)	127 (1.8)	1.00
	Yes	2360 (19.6)	71 (3.0)	1.30 (0.97 to 1.75)	1870 (21.3)	54 (2.9)	1.37 (0.97 to 1.92)
Time of delivery††	On time	11 154 (92.8)	207 (1.9)	1.00	8116 (92.5)	153 (1.9)	1.00
	Early	358 (3.0)	36 (10.1)	5.00 (3.37 to 7.42)	275 (3.1)	21 (7.6)	3.46 (2.09 to 5.74)
	After due date	503 (4.2)	13 (2.6)	1.27 (0.71 to 2.25)	382 (4.4)	7 (1.8)	0.86 (0.40 to 1.87)
Any antenatal care check-up during pregnancy‡‡	Yes	9946 (82.8)	198 (2.0)	1.00	7414 (84.5)	148 (2.0)	1.00
	No	2069 (17.2)	58 (2.8)	1.51 (1.11 to 2.04)	1359 (15.5)	33 (2.4)	1.28 (0.87 to 1.89)
Mother consumed 90 or more iron folic acid tablets§§	Yes	2043 (17.0)	36 (1.8)	1.00	1658 (18.9)	26 (1.6)	1.00
	No	9972 (83.0)	220 (2.2)	1.15 (0.80 to 1.65)	7115 (81.1)	155 (2.2)	1.32 (0.87 to 2.02)
Reported use of antiseptic for cord care¶¶	Antiseptic applied	5969 (49.7)	104 (1.7)	1.00	4781 (54.5)	81 (1.7)	1.00
	Nothing applied	5481 (45.6)	145 (2.7)	1.53 (1.18 to 1.99)	3739 (42.6)	97 (2.6)	1.52 (1.12 to 2.06)
	Harmful substance used	565 (4.7)	7 (1.2)	0.72 (0.33 to 1.57)	253 (2.9)	3 (1.2)	0.69 (0.21 to 2.19)
Delayed bathing of neonate (after 2 days)***	Yes	6012 (50.0)	76 (1.3)	1.00	4761 (54.3)	60 (1.3)	1.00
	No	6003 (50.0)	180 (3.0)	2.39 (1.81 to 3.15)	4012 (45.7)	121 (3.0)	2.38 (1.73 to 3.26)
Neonate received Kangaroo care (skin to skin contact) †††	Yes	4017 (33.5)	76 (1.9)	1.00	3164 (36.2)	59 (1.9)	1.00
	No	7955 (66.5)	179 (2.3)	1.05 (0.79 to 1.39)	5567 (63.8)	121 (2.2)	1.08 (0.79 to 1.49)
Early breast feeding of neonate (immediately/within 1 hour) ‡‡‡	Yes	5595 (46.6)	88 (1.6)	1.00	4207 (48.0)	69 (1.6)	1.00
	No	6420 (53.4)	168 (2.6)	1.54 (1.17 to 2.01)	4566 (52.0)	112 (2.5)	1.43 (1.04 to 1.95)
Mother received postnatal check-up within 2 weeks§§§	Yes	4154 (34.6)	72 (1.7)	1.00	4121 (47.0)	72 (1.8)	1.00
	No	7861 (65.4)	184 (2.3)	1.41 (1.04 to 1.93)	4652 (53.0)	109 (2.3)	1.35 (0.99 to 1.84)

*χ^2^ test for significance: p=0.009 for overall and 0.006 for institutional delivery.

†χ^2^ test for significance: p=0.590 for overall and 0.785 for institutional delivery.

‡χ^2^ test for significance: p=0.071 for overall and 0.285 for institutional delivery.

§Data missing for 12 live births; χ^2^ test for significance: p=0.084 for overall and 0.134 for institutional delivery.

¶χ^2^ test for significance: p=0.283 for overall.

**χ^2^ test for significance: p=0.020 for overall and 0.303 for institutional delivery.

††χ^2^ test for significance: p<0.001 for both; time of delivery as defined by the respondent.

‡‡χ^2^ test for significance: p=0.990 for overall and 0.657 for institutional delivery.

§§χ^2^ test for significance: p=0.205 for overall and 0.115 for institutional delivery.

¶¶χ^2^ test for significance: p=0.003 for overall and 0.008 for institutional delivery; harmful substances include sindoor, ghee and cow dung.

***χ^2^ test for significance: p<0.001 for both.

†††Data missing for 43 live births; χ^2^ test for significance: p=0.200 for overall and 0.329 for institutional delivery.

‡‡‡χ^2^ test for significance: p<0.001 for overall and p=0.007 for institutional delivery.

§§§χ^2^ test for significance: p=0.028 for overall and 0.050 for institutional delivery.

## Discussion

In this large population-based study, mortality was lower among neonates for whom use of antiseptic on the cord was reported compared with those who did not in a representative sample of live births in the Indian state of Bihar which has high neonatal mortality. This finding remained true for live births in health facilities as well. Gentian violet was the antiseptic of choice for the majority who reported use of antiseptic for cord care.

Even though the skilled birth attendance guidelines in India recommend dry cord care for all births,^16^ half of the live births reported use of antiseptic on the cord in this study population. WHO recommends dry cord care for institutional births.^13^ We, however, found antiseptic cord care to have a beneficial influence on neonatal mortality among institutional births also. The use of antiseptic for cord care was reported significantly more for live births in private health facilities as compared with public facilities in this study, and for deliveries performed by a qualified healthcare provider. The use of antiseptic for cord care was significantly lower in home births. However, deliveries performed by a qualified healthcare provider at home reported a higher use of antiseptic. Interestingly, reporting of antiseptic use among deliveries performed by doctors was lower than among those performed by a nurse or ANM, which is likely a reflection of better awareness among doctors about the official dry cord care recommendation.

After adjusting for the potential risk factors for neonatal mortality, we found the use of antiseptic for cord care, predominantly gentian violet, to be significantly protective for newborn survival. Gentian violet was an important topical antiseptic until being superseded by modern drugs, and was listed in the WHO Essential Drug List for children until 2009.^23^ It is still commonly used in the Indian healthcare settings, and is included in the Indian Public Health guidelines for various levels of health facilities.^24^ With the evidence of a 50% reduction in the incidence of omphalitis and a 12% reduction in neonatal mortality with chlorhexidine from community-based randomised controlled trials,^6–11^ chlorhexidine is currently preferred as it is active against aerobic and anaerobic organisms, and it is also currently included in the WHO Essential Drug List.^25^ But there is some uncertainty as to the effect of chlorhexidine in hospital settings on neonatal mortality.^7^ ^10^ ^11^ Use of chlorhexidine has also been found to be cost-effective as compared with other antiseptics in hospital settings for surgical infections. Application of chlorhexidine on the cord after birth is not yet recommended by health authorities in India. Not surprisingly, therefore, the use of chlorhexidine was negligible in our study population and gentian violet was the antiseptic of choice.

These large-scale state-wide representative data suggest that gentian violet application on the cord after birth could be potentially beneficial for newborn health in this setting. We are not aware of trials designed to assess the effect of gentian violet for cleansing of the umbilical cord. A study in the north Indian state of Uttar Pradesh, neighbouring Bihar, on births in 2004–2005 reported lower neonatal mortality with clean cord care (defined as clean instrument to cut cord, clean thread to tie cord, and antiseptic or nothing applied to cord), but did not report the distinction between antiseptic and dry cord care.^26^ Additional observational studies would be useful to better understand use of gentian violet for cord care, which is readily available for use in India and in other low-resource settings.

The associations of variables other than use of antiseptic for cord care with neonatal mortality were generally similar in this analysis to those reported by us in the survey of 2012 in this population with a few differences.^19^ As in the previous survey, the current survey of 2014 again highlighted the significant association of postnatal care-related variables and younger maternal age with neonatal mortality in this population.^19^ Addressing the health of adolescent girls which can impact pregnancy and health of the newborn, in addition to delaying the age at marriage for girls need immediate attention.^27^ Postnatal care has until now received less attention as part of maternal and child health interventions in India, which is likely to change with the adoption of the New Born Action Plan.^28^

Even though significantly lower use of antiseptic was reported for preterm babies, the use of antiseptic was beneficial among these babies. Given that small immature babies are likely to be more vulnerable, appropriate care for such babies is necessary to reduce mortality. Community trials of chlorhexidine have shown that the incidence of omphalitis and neonatal mortality can be reduced in preterm newborns,^7^ and salicylic sugar powder has been shown to be effective with premature infants in intensive care unit.^29^ With preterm delivery being the most significant predictor of neonatal mortality in our study population, the potential benefit of antiseptic cord care should be explored further in this group. We were unable to assess the association of low birth weight with use of antiseptic for cord care or neonatal mortality as this information was missing for 44.1% of the neonates in this study. Such high proportion of missingness for low birth weight has also been reported in the Annual Health Survey for Bihar (57.1%).^30^ One of the encouraging findings is the very low use of harmful substances for cord care in this population (4.7%), suggesting that relevant cord care messages have percolated in the community. The use of potentially harmful substances on the cord was three times more common among home births where most of the deliveries were conducted by unqualified healthcare providers. A focussed understanding and approach would be needed to address this at the community level.^31^ ^32^

Our study has some limitations. The information about cord care was documented as reported by respondents and is not based on direct observation. We think that this reporting is generally reliable given that majority of those who reported antiseptic cord care reported the use of gentian violet, which was identified by the respondents as *neeli dava* (name in local language) that leaves a distinct purplish-blue colour after application. The timing of antiseptic use, who applied the antiseptic and the time taken for cord separation were not documented. Importantly, data on any illness preceding death, cause of death or incidence of omphalitis were not available. Finally, it is not possible to account for all the potential confounders in a cross-sectional survey. Nonetheless, the significant beneficial association of antiseptic use with neonatal mortality found in this study, after adjusting for several other variables that are associated with neonatal mortality in this population, is relevant.

In summary, these large-scale population-based cross-sectional data suggest a significant association of the use of antiseptic for cord care with lower all-cause neonatal mortality in an Indian state with a relatively high rate of neonatal deaths. This beneficial association was found for births at home and at health facilities, and predominately with the application of the readily available gentian violet. Although these are findings from a cross-sectional survey and not a randomised trial, these highlight the need to explore further the protective association of gentian violet for neonatal mortality in the developing country setting. Even though the official recommendation is for dry cord care in India, use of antiseptic for cord care was reported for half of the live births. In the background of current reluctance in India to introduce chlorhexidine for cord care, a higher use of the readily available gentian violet may potentially help in reducing neonatal mortality.
